# Perceived neighborhood social cohesion and cervical and breast cancer screening utilization among U.S.-born and immigrant women

**DOI:** 10.3934/publichealth.2022039

**Published:** 2022-07-11

**Authors:** Quynh Nhu (Natasha) B. La Frinere-Sandoval, Catherine Cubbin, Diana M. DiNitto

**Affiliations:** Steve Hicks School of Social Work, The University of Texas at Austin

**Keywords:** breast cancer, cervical cancer, mammogram, Pap test, preventive screening, immigrant, neighborhood social cohesion

## Abstract

Research suggests that factors beyond the individual level, such as neighborhood-level factors, warrant further investigation in explaining preventive screening utilization disparities. In addition, research shows that immigrant women, especially recent immigrants, are less likely than U.S.-born women to utilize preventive screenings. Our study examined the relationship between perceived neighborhood social cohesion and breast and cervical cancer screening utilization among U.S.-born and immigrant women. Data came from the 2018 National Health Interview Survey (NHIS). The sample for this study included 7801 women ages 21−64 without a hysterectomy. Of them, 1477 (19%) reported being born outside the United States. Logistic regression was used to examine associations of perceived neighborhood social cohesion and sociodemographic factors with the odds of screening by nativity status. Though we found no link between neighborhood social cohesion and Papanicolaou (Pap) test or mammogram utilization, our findings contribute to understanding sociodemographic barriers to and facilitators of preventive screening utilization among immigrant and U.S.-born women. Most importantly, racial/ethnic and socioeconomic disparities in Pap tests and mammogram utilization were evident among immigrant women. The disparities we identified indicate the need to target prevention messages and tailor interventions to address each group's sociodemographic characteristics and needs. Our findings also support the need to expand health insurance so that all women are covered.

## 1. Introduction

Breast cancer is the world's most common type of cancer, with an estimated 2.3 million diagnosed cases and 685,000 deaths from this illness in 2020 [1]. Cervical cancer is the fourth most prevalent cancer among women globally, with approximately 604,000 cases diagnosed and 342,000 deaths in 2020 [2]. Numerous studies from different countries, including the United States, Canada, and Australia, have consistently found immigrant women to be at higher risk for underutilizing screenings for cervical cancer [3]–[5] and breast cancer [6],[7] than native-born women. In 2018, the number of immigrants living in the United States (U.S.) reached a record 44.8 million or 13.7% of the population [8]. Within the Hispanic and Asian ethnic groups, the proportion of immigrants is higher, comprising one third and nearly two thirds of these groups, respectively [9],[10]. Immigrant women, especially recent immigrants, are less likely than U.S.-born women to utilize preventive screenings [11]–[13]. Watson et al. [14] found that approximately one third of immigrant women with less than ten years of U.S. residency did not meet recommended preventive screening guidelines. Additionally, immigrant women had significantly lower mammogram screening utilization rates within the preceding two years than U.S.-born women (68.8% and 73.0%, respectively) [13]. Although cervical and breast cancer are considered highly treatable with early detection, research consistently shows that immigrant women have lower screening rates than U.S.-born women [13],[15].

Previous studies have predominantly focused on individual-level risk factors predisposing immigrant women to postpone healthcare, such as language and cultural barriers, lack of knowledge about cervical cancer screening [16], shorter length of U.S. residence [17], and lack of health insurance [3]. Several studies also noted that immigrant women had lower rates of screening utilization than U.S.-born women even when potential variations in demographic factors, healthcare access, and health status were controlled [11].

Recent research suggests that factors beyond the individual level, such as neighborhood-level factors, warrant further investigation in explaining disparities in screening utilization among immigrant groups [18]. While most neighborhood research has examined the deleterious effects of negative neighborhood factors on population health, recent studies suggest that positive neighborhood-level factors, such as neighborhood social cohesion, can have beneficial effects on health outcomes [18]. “Social cohesion” has been defined as “the perceived degree of connectedness among groups in society” [19] and “willingness to intervene for the common good” [20]. Among the features of neighborhood social cohesion are feelings of belongingness and shared trust among neighbors [20].

Kim and Kawachi [18] hypothesized that environmental factors such as neighborhood social cohesion may increase preventive health services use by expanding health services information dissemination, providing social and emotional support, promoting shared capability to petition for resources, and strengthening and sustaining healthy behavior norms in the community. However, results from the handful of pioneering studies examining the effects of positive neighborhood characteristics on preventive services are inconsistent. Using data from The Health and Retirement Survey's nationally representative sample (n = 7168 respondents age 50+ in 2006), Kim and Kawachi [18] found that higher neighborhood social cohesion was linked to increased use of every type of preventive care studied (influenza vaccinations, cholesterol tests, mammograms, and Papanicolaou [Pap] tests) except prostate screenings. Dean et al. [21] used multilevel analysis to explore African-American women's mammography use in Philadelphia. Defining neighborhood social capital as social cohesion, collective efficacy, and social participation, they did not find a significant relationship between social cohesion and mammography in multivariable analyses controlling for individual and community-level factors. More recently, Ali et al. [22] assessed neighborhood social cohesion's effects on preventive screenings for hypertension, high cholesterol, diabetes, and depression in Asian American communities in New York City. Using both a categorical and a continuous measure, they found that overall neighborhood social cohesion was associated with increased utilization of cholesterol screening for all groups, but results for diabetes and depression screening varied among ethnic groups. For example, while neighborhood social cohesion was not significantly associated with diabetes or depression screening among East Asian and Southeast Asian Americans, it was significant for South Asian Americans.

In the United States, disparities in cervical and breast cancer screening between U.S.-born women and the rapidly growing population of immigrant women call for further examination of social factors, including community and neighborhood factors, that in addition to individual level factors (e.g., income, race/ethnicity, education), may be associated with health behaviors, such as preventive care utilization. Previous research has highlighted both individual and structural factors as important social determinants of health and underlined their relevance for influencing efforts to encourage cancer screening utilization [23]. The Social Determinants of Health conceptual framework [24] illustrates the means by which social, economic and political forces contribute to the socioeconomic stratification of populations based on various factors such as income, gender, employment, education level, marital status, and race/ethnicity. One's socioeconomic status influences these health status drivers since those with low socioeconomic status are generally more susceptible to situations that are harmful to their health. Guided by this conceptual framework, we examined the extent to which neighborhood social cohesion and sociodemographic characteristics influence screening utilization among immigrant and U.S.-born women.

We hypothesized that (H1) perceived neighborhood social cohesion will be associated with increased preventive service utilization; (H2) perceived neighborhood cohesion's impact on screening utilization will differ between U.S.-born and immigrant groups; and (H3) racial/ethnic disparities will emerge in screening utilization among both U.S.-born and immigrant groups even after controlling for sociodemographic characteristics.

## 2. Materials and methods

### 2.1. Data source and study sample

Data came from the 2018 National Health Interview Survey (NHIS), a nationally representative, cross-sectional household interview survey of the U.S. civilian, non-institutionalized population. NHIS's primary goal is to continuously monitor the U.S. population's health through large scale data collection across a wide spectrum of health issues [25]. The overall sample for this population-based study was the 7801 women ages 21–64 without a hysterectomy. Of them, 7722 (99%) reported Pap test data. The overall sample also included 4211 women ages 40–64 without a hysterectomy, of whom 4087 (78%) reported mammogram data. Of the 7801 women, 1477 (19%) reported being born outside the United States and are considered immigrants. Since virtually all adults age 65 and older in the United States are eligible for Medicare, a federal health insurance program, women in this age group were excluded from the study due to insufficient variance in their insurance status. Participants who identified as belonging to a racial group other than Non-Hispanic Asian, Non-Hispanic Black, Hispanic, or Non-Hispanic White were excluded from our study sample because their numbers were too small for multivariable statistical analyses. The University of Texas at Austin Institutional Review Board reviewed this study's protocol and determined that this is not research involving human subjects and is therefore exempt from IRB oversight.

### 2.2. Measures

Dependent variables were Pap test and mammogram utilization meeting American Cancer Society (ACS) or U.S. Preventive Services Task Force (USPSTF) guidelines. USPSTF recommends that women ages 21−65 of average risk have a Pap test every three years [26]; therefore, we gauged Pap test screening utilization using NHIS's query about *“Most recent Pap test, time categories”* excluding cases that reported having a hysterectomy. Those reporting that they were screened *“a year ago or less” “more than 1 year but not more than 2 years”* or *“more than 2 years but not more than 3 years”* were coded as “Yes”; the rest were coded as “No” For mammograms, recommendations are that women ages 40 and older be screened every year [27] or every two years [28]. Using NHIS's query about having had a mammogram “*a year ago or less*” or “*more than 1 year but not more than 2 years*” we coded those in this age group who responded affirmatively to either query as “Yes” and those who chose another answer as “No”.

The independent variable was perceived neighborhood social cohesion. NHIS queried participants on various neighborhood factors by asking whether they agree or disagree with each of the following four statements using a scale from 1 (definitely agree) to 4 (definitely disagree): 1) “People in this neighborhood help each other out”; 2) “There are people I can count on in this neighborhood”; 3) “People in this neighborhood can be trusted”; and 4) “This is a close-knit neighborhood.” In prior studies, these four items were used to form a neighborhood social cohesion scale that demonstrated high internal consistency (Cronbach's alpha 0.93) [20],[29]. Each social cohesion scale item is first reverse coded so that a higher score indicates higher social cohesion; the value of each of the four items is then summed to form a continuous variable with scores ranging from 4 to 16. In our study, we then standardized the summed scores so that in the multivariable analyses the odds ratios indicate neighborhood social cohesion scores as standard deviations from the mean [18]. We imputed any missing or not reported cases for each question separately as the mean of the reported cases for that specific question.

We selected control variables based on previous cancer screening utilization research [15],[30],[31]. Sociodemographic variables were age (years), marital status (divorced/separated/widowed, never married, married/cohabiting), and race/ethnicity (Asian, Black, Hispanic, White). Socioeconomic status (SES) variables included education (less than high school degree, high school degree, some college, or college degree), employment status (worked last week or not), family income as a share of the federal poverty level (FPL) (FPL <100%, 100–199%, 200–399%, >400%), and health insurance status (uninsured or insured). The Census Bureau defines threshold levels of income based on family size (one or more) and age, adjusted for inflation. This base income level is uniform throughout the United States. Total family income is calculated by summing the incomes of all members of the family. The income for an individual or family can be normalized by transforming it to a percentage of the FPL. Individuals or families with income below 100% FPL are considered the lowest income group and those at or above 400% FPL, are the highest income group [32]. Nativity was defined as U.S.-born vs. immigrant, and acculturation level among immigrants was defined as years living in the United States (less than 5 years, 5–less than 10 years, 10–15 years, and >15 years).

### 2.3. Analytic plan

First, we examined the distribution of all variables overall and then stratified by nativity. To test study hypotheses, we used logistic regression to compute odds ratios and 95% confidence intervals (CI). We examined three models: (1) an unadjusted model (with no control variables), (2) a model adjusted for sociodemographic characteristics (age, race/ethnicity, marital status, insurance coverage, years living in the United States, and perceived neighborhood cohesion), and (3) a full model that included SES variables (education, employment status, income) in addition to all the sociodemographic variables from the second model. All analyses were weighted to account for NHIS's complex sampling design. We included a squared term for perceived neighborhood social cohesion to test for a non-linear relationship with the dependent variables Pap test and mammogram utilization. To examine income's relationship, we used NHIS's multiply imputed income data files. To integrate these data, we used SUDAAN's multiple imputation functions, along with a SAS macro to create five separate analysis files, one for each version of imputed income data. SAS 9.4 and SAS-callable SUDAAN were used for all analyses. Odds ratios for the age variable are expressed as the incremental increase in odds for each additional year of age of the subject, holding other variables constant.

## 3. Results

### 3.1. Participants' characteristics

As Table 1 shows, immigrant and U.S.-born women differed significantly on all characteristics except age and mammogram utilization. Foreign-born women had higher proportions of those who were Asian and Hispanic, married/cohabitating, uninsured, and unemployed, and they had less education and lower income and perceived neighborhood social cohesion than U.S.-born women. Among immigrants, 64% had lived in the United States over 15 years while 12%, 11%, and 12% had lived in the U.S for less than 5 years, 5 to less than 10 years, and 10 to 15 years, respectively. Immigrant women had a lower rate of Pap test utilization (76%) than U.S.-born women (82%).

As Table 2 shows, unadjusted model results revealed some similarities between immigrant and U.S.-born women with regard to Pap test utilization. Those who were never married or unemployed, had lower income, or lacked health insurance had lower odds of having had a Pap test compared with their reference groups. Among immigrants, those who had lived in the United States for less than 10 years also had lower odds than those living in the United States for more than 15 years. Among U.S.-born women, those who were Black, previously married, or had less than a college education also had lower odds. Neighborhood social cohesion was not associated with Pap test utilization among either immigrant or U.S.-born women.

**Table 1.** Descriptive statistics for women ages 21–64, National Health Interview Survey, 2018, N = 7722.

	Immigrant 1477 (19%)	U.S.-Born 6324 (81%)	T or Chi-Square Statistic	Significance Level
Age (mean)	42.3 (0.4)	40.5 (0.2)	1.77	0.0769
Race/Ethnicity			2616.00	0.0001
Asian	351 (27%)	116 (2%)		
Black	138 (11%)	894 (14%)		
Hispanic	700 (47%)	576 (11%)		
White	274 (16%)	4638 (72%)		
Marital Status			63.54	0.0001
Divorced/Separated/Widowed	271 (13%)	1280 (13%)		
Never Married	267 (15%)	1696 (26%)		
Married/Cohabiting	937 (72%)	3335 (61%)		
Education			356.63	0.0001
Less than high school degree	304 (20%)	386 (6%)		
High school degree	304 (22%)	1216 (20%)		
Some college	291 (20%)	2077 (33%)		
College graduate	569 (38%)	2631 (41%)		
Employment Status			28.64	0.0001
Did not work last week	548 (39%)	1895 (30%)		
Worked last week	927 (61%)	4427 (70%)		
Income (% of Federal Poverty Level)			101.33	0.0001
<100%	293 (17%)	864 (11%)		
100%–199%	341 (24%)	1,017 (15%)		
200%–299%	212 (15%)	944 (15%)		
300%–399%	163 (11%)	850 (14%)		
>400%	468 (32%)	2649 (45%)		
Health Insurance			152.94	0.0001
Not covered	307 (21%)	593 (9%)		
Covered	1164 (79%)	5708 (91%)		
Years living in U.S.				
<5 years	182 (12%)			
5–less than 10 years	141 (11%)			
10–15 years	183 (12%)			
>15 years	950 (64%)			
Perceived neighborhood social cohesion			
	11.9 (0.1)	12.4 (0.1)	−5.13	0.0001
Pap-test last 3 years (ages 21–64)			21.31	0.0001
Yes	1120 (76%)	5120 (82%)		
No	348 (24%)	1170 (18%)		
Mammogram last 2 years (ages 40–64)			2.04	0.1537
Yes	514 (62%)	2168 (66%)		
No	312 (38%)	1093 (34%)		

Racial/ethnic disparities emerged in the sociodemographic models for Pap test utilization. Both U.S.-born and immigrant Hispanic women and U.S.-born Black women had higher odds of having a Pap test than their White counterparts. Other results were similar to the unadjusted models. An additional racial/ethnic disparity emerged in the full model with immigrant Asian women having lower odds of Pap test use than immigrant White women. Other results were similar to the unadjusted and sociodemographic models, except that for U.S.-born women, being previously married or unemployed were no longer statistically significant. For both groups, older age was associated with lower odds of getting a Pap test.

Table 3 presents odds ratios and confidence intervals for mammogram utilization (for women ages 40–64). 

In the unadjusted models, among immigrant women, those who had less than a high-school education, or income lower than 200% had significantly lower odds of mammogram utilization, while among U.S.-born women, those who were Asian, were previously or never married, had high-school degree or less education, were unemployed, or had income lower than 400% had lower odds of having a mammogram.

As with Pap test utilization, racial/ethnic disparities emerged in the sociodemographic model. Both U.S.-born and immigrant women who lacked insurance had lower odds of mammogram utilization. Immigrant Black women and Asian women had higher odds of mammogram utilization than their White counterparts. Among immigrant women, those who had lived in the United States for less than 10 years had lower odds of having a mammogram than those living in the United States for more than 15 years. Among U.S.-born women, those who were Asian and those who never married had lower odds of mammogram utilization. Perceived social cohesion was associated with higher odds of mammogram utilization among U.S.-born women (OR = 1.63, CI = 1.02, 2.60).

Most of the significant factors remained in the full model. For both immigrant and U.S.-born groups, older age was associated with higher odds of mammogram utilization, while not having insurance coverage and income less than 200% was associated with lower odds. Among immigrants, Black and Asian (compared with White) women had *higher* odds of mammogram utilization. Those who had lived in the United States for less than 10 years had lower odds of having mammogram utilization than those living in the United States for more than 15 years. Continuing the same trend, among U.S.-born women, Black women had higher odds of mammogram utilization compared to their White counterparts. Those who had less than a high school degree had lower odds of mammogram utilization. For both groups, perceived social cohesion had no effect.

**Table 2.** Odds ratios of Pap test utilization, NHIS, U.S., 2018, N = 7722.

	Unadjusted Models				Sociodemographic Models				Full Models			
	Immigrant		U.S.-Born		Immigrant		U.S.-Born		Immigrant		U.S.-Born	
	O.R.	95% C.I.	O.R.	95% C.I.	O.R.	95% C.I.	O.R.	95% C.I.	O.R.	95% C.I.	O.R.	95% C.I.
Age	1.01	[1.00, 1.03]	0.99	[0.98, 0.99]	0.99	[0.97, 1.00]	0.98	[0.97, 0.99]	0.99	[0.97, 1.00]	0.98	[0.97, 0.99]
Race/Ethnicity												
Asian	0.69	[0.45, 1.06]	0.95	[0.60, 1.50]	0.68	[0.43, 1.08]	0.88	[0.56, 1.37]	0.62	[0.39, 0.99]	0.76	[0.48, 1.20]
Black	0.92	[0.53, 1.60]	1.77	[1.39, 2.26]	1.19	[0.67, 2.12]	2.45	[1.87, 3.20]	1.47	[0.80, 2.68]	3.07	[2.35, 4.00]
Hispanic	1.04	[0.68, 1.59]	1.15	[0.91, 1.47]	1.62	[1.05, 2.48]	1.30	[1.00, 1.70]	2.12	[1.34, 3.35]	1.52	[1.16, 2.00]
White	1.00		1.00		1.00		1.00		1.00		1.00	
Marital Status												
Divorced/Separated/Widowed	1.03	[0.71, 1.50]	0.60	[0.50, 0.71]	1.00	[0.66, 1.51]	0.71	[0.59, 0.85]	1.07	[0.70, 1.64]	0.85	[0.70, 1.03]
Never Married	0.44	[0.32, 0.60]	0.56	[0.48, 0.67]	0.43	[0.30, 0.62]	0.43	[0.36, 0.52]	0.42	[0.28, 0.62]	0.48	[0.39, 0.59]
Married/Cohabiting	1.00		1.00		1.00		1.00		1.00		1.00	
Education												
Less than high school degree	0.69	[0.45, 1.07]	0.33	[0.25, 0.44]					0.74	[0.44, 1.24]	0.45	[0.33, 0.62]
High school degree	0.73	[0.50, 1.07]	0.45	[0.37, 0.55]					0.68	[0.42, 1.11]	0.56	[0.45, 0.70]
Some college	0.76	[0.52, 1.09]	0.62	[0.52, 0.75]					0.76	[0.50, 1.14]	0.70	[0.57, 0.85]
College graduate	1.00		1.00						1.00		1.00	
Employment Status												
Did not work last week	0.67	[0.51, 0.87]	0.72	[0.63, 0.83]					0.72	[0.53, 0.98]	0.96	[0.81, 1.13]
Worked last week	1.00		1.00						1.00		1.00	
Income (% of Federal Poverty Level)										
<100%	0.42	[0.28, 0.64]	0.39	[0.32, 0.48]					0.62	[0.35, 1.10]	0.52	[0.39, 0.69]
100%–199%	0.54	[0.36, 0.80]	0.48	[0.39, 0.59]					0.59	[0.36, 0.97]	0.60	[0.47, 0.78]
200%–299%	0.71	[0.44, 1.16]	0.56	[0.45, 0.70]					0.71	[0.42, 1.19]	0.63	[0.50, 0.80]
300%–399%	1.05	[0.60, 1.85]	0.71	[0.56, 0.91]					1.22	[0.66, 2.23]	0.76	[0.58, 0.98]
≥400%	1.00		1.00						1.00		1.00	
Health Insurance Coverage												
Not covered	0.43	[0.30, 0.61]	0.34	[0.28, 0.42]	0.32	[0.22, 0.46]	0.34	[0.28, 0.41]	0.37	[0.26, 0.54]	0.42	[0.35, 0.52]
Covered	1.00		1.00		1.00		1.00		1.00		1.00	
Years Living in U.S.												
<5 years	0.37	[0.26, 0.53]			0.42	[0.28, 0.65]			0.45	[0.29, 0.69]		
5–less than 10 years	0.59	[0.38, 0.93]			0.59	[0.36, 0.98]			0.59	[0.37, 0.96]		
10–15 years	1.10	[0.71, 1.69]			1.13	[0.71, 1.81]			1.19	[0.73, 1.93]		
>15 Years	1.00				1.00				1.00			
Perceived Neighborhood Social Cohesion											
	1.99	[0.90, 4.39]	0.75	[0.51, 1.10]	0.74	[0.33, 1.67]	1.27	[0.86, 1.86]	0.74	[0.32, 1.70]	1.04	[0.70, 1.54]
Perceived Neighborhood Social Cohesion Squared										
	0.59	[0.27, 1.30]	1.44	[0.99, 2.08]	1.57	[0.69, 3.58]	0.86	[0.57, 1.27]	1.56	[0.67, 3.64]	0.98	[0.65, 1.48]

**Table 3.** Odds ratios of mammogram utilization, NHIS, U.S., 2018, N = 4087

	Unadjusted Model				Sociodemographic Models				Full Models			
	Immigrant		U.S.-Born		Immigrant		U.S.-Born		Immigrant		U.S.-Born	
	O.R.	95% C.I.	O.R.	95% C.I.	O.R.	95% C.I.	O.R.	95% C.I.	O.R.	95% C.I.	O.R.	95% C.I.
Age	1.05	[1.03, 1.08]	1.04	[1.03, 1.05]	1.04	[1.02, 1.07]	1.04	[1.03, 1.06]	1.05	[1.02, 1.07]	1.05	[1.04, 1.06]
Race/Ethnicity												
Asian	1.01	[0.69, 1.47]	0.57	[0.42, 0.77]	1.75	[1.13, 2.70]	0.71	[0.51, 0.98]	2.87	[1.70, 4.85]	0.80	[0.57, 1.13]
Black	1.32	[0.73, 2.41]	0.87	[0.67, 1.13]	2.15	[1.12, 4.11]	1.06	[0.80, 1.40]	2.83	[1.38, 5.80]	1.31	[1.00, 1.72]
Hispanic	0.87	[0.57, 1.33]	1.24	[0.49, 3.13]	0.92	[0.59, 1.42]	1.37	[0.56, 3.35]	0.97	[0.62, 1.51]	1.11	[0.45, 2.76]
White	1.00		1.00		1.00		1.00		1.00		1.00	
Marital Status												
Divorced/Separated/Widowed	0.96	[0.67, 1.36]	0.80	[0.67, 0.96]	0.75	[0.51, 1.11]	0.84	[0.69, 1.01]	0.93	[0.62, 1.42]	1.02	[0.83, 1.25]
Never Married	0.75	[0.44, 1.27]	0.58	[0.46, 0.73]	0.67	[0.37, 1.20]	0.64	[0.49, 0.83]	0.79	[0.42, 1.47]	0.77	[0.59, 1.01]
Married/Cohabiting	1.00		1.00		1.00		1.00		1.00		1.00	
Education												
Less than high school degree	0.50	[0.34, 0.74]	0.39	[0.29, 0.53]					0.69	[0.41, 1.16]	0.69	[0.48, 0.99]
High school degree	0.69	[0.47, 1.02]	0.59	[0.48, 0.74]					0.72	[0.43, 1.18]	0.76	[0.59, 0.98]
Some college	0.89	[0.57, 1.37]	0.68	[0.56, 0.82]					0.98	[0.60, 1.60]	0.76	[0.61, 0.95]
College graduate	1.00		1.00						1.00		1.00	
Employment Status												
Did not work last week	0.87	[0.66, 1.16]	0.74	[0.63, 0.87]					1.11	[0.79, 1.57]	0.83	[0.68, 1.00]
Worked last week	1.00		1.00						1.00		1.00	
Income (% of Federal Poverty Level)												
<100%	0.32	[0.21, 0.51]	0.28	[0.21, 0.36]					0.35	[0.18, 0.65]	0.42	[0.30, 0.58]
100%–199%	0.45	[0.30, 0.68]	0.44	[0.34, 0.56]					0.48	[0.28, 0.84]	0.60	[0.44, 0.82]
200%–299%	0.64	[0.37, 1.11]	0.63	[0.49, 0.81]					0.62	[0.33, 1.14]	0.77	[0.59, 1.01]
300%–399%	0.56	[0.32, 1.00]	0.72	[0.56, 0.94]					0.56	[0.29, 1.09]	0.79	[0.60, 1.04]
≥400%	1.00		1.00						1.00		1.00	
Health Insurance Coverage												
Not covered	0.26	[0.17, 0.39]	0.23	[0.18, 0.30]	0.23	[0.14, 0.36]	0.23	[0.17, 0.31]	0.27	[0.16, 0.43]	0.28	[0.21, 0.37]
Covered	1.00		1.00		1.00		1.00		1.00		1.00	
Years Living in U.S.												
<5 years	0.36	[0.18, 0.73]			0.48	[0.24, 0.95]			0.49	[0.24, 1.01]		
5–less than 10 years	0.36	[0.17, 0.77]			0.36	[0.16, 0.84]			0.41	[0.17, 0.97]		
10–15 years	0.79	[0.47, 1.32]			0.91	[0.53, 1.57]			1.03	[0.59, 1.79]		
>15 Years	1.00				1.00				1.00			
Perceived Neighborhood Social Cohesion												
	0.68	[0.29, 1.63]	1.70	[1.09, 2.65]	0.81	[0.32, 2.08]	1.63	[1.02, 2.60]	0.80	[0.30, 2.12]	1.27	[0.79, 2.04]
Perceived Neighborhood Social Cohesion Squared												
	1.73	[0.71, 4.20]	0.69	[0.44, 1.07]	1.37	[0.53, 3.54]	0.69	[0.43, 1.10]	1.37	[0.51, 3.69]	0.83	[0.52, 1.33]

## 4. Discussion

Contrary to our expectations, neighborhood social cohesion was not significantly associated with preventive cancer screenings in any models for immigrant or U.S.-born women. As mentioned, prior studies on the relationship between preventive screenings and neighborhood social cohesion have produced differing results. Several studies found that living in more cohesive neighborhoods was associated with higher preventive services utilization [18],[22],[33]. Dean et al. [21] did not find a significant association and suggested that the dissemination of shared knowledge within a highly cohesive neighborhood may not foster an effect strong enough for residents to overcome obstacles to utilizing these types of screening services. These obstacles may include limited healthcare availability, inability to access healthcare resources, and other disadvantages community members face [21]. Although we did not find a link between neighborhood social cohesion and increased Pap-test and mammogram utilization among women using NHIS data, our findings contribute to a better understanding of barriers and factors that facilitate preventive screening utilization among immigrant and U.S-born women in a nationally representative sample.

Disparities in preventive service utilization across socioeconomic classifications are well documented in the research literature [14],[34]. Individual socioeconomic factors were strong predictors of Pap test and mammogram screening utilization in our study given that both socially disadvantaged U.S.-born and immigrant women had lower odds of Pap test and mammogram use. Consistent with prior studies [3],[34],[35], we also found that lack of health insurance was an important predictor of lower Pap-test and mammogram screening utilization among both U.S.-born and immigrant women.

The sociodemographic model also revealed racial/ethnic disparities in Pap test utilization that are contrary to commonly reported trends. For example, like some previous studies, we found that among both U.S.-born and immigrant groups, Hispanic women had higher odds of Pap test use than White women [36] and that among the U.S.-born, Black women had higher odds of Pap test use than White women [37]. Any obstacles these groups may have faced in obtaining preventive services might have been overcome through increased access and outreach. For example, national and regional programs and initiatives have been launched that are specifically tailored to racial minority populations in an effort to reduce disparities and improve cancer screening among these groups [37]. In the full model for immigrant women, consistent with other studies [11],[38], another racial/ethnic disparity emerged in that Asian women had lower odds of Pap test screenings than their White counterparts. However, pooling NHIS data from 4 years (2005, 2008, 2013, 2015), Endeshaw et al. [39] found that the likelihood of having received a Pap test within 3 years for immigrant Southeast Asian women was comparable to U.S.-born women. Although those results suggest that Pap test utilization has increased in recent years among Asian immigrant women, our study indicates that this group remains at risk of underutilization of cervical cancer preventive screenings. In comparing Asian immigrants to White immigrants, we found that disparities in utilization persist.

Our finding that never married women had lower Pap-test utilization rates than married or cohabitating women deserves attention. Clark et al. [40] found that single women reported lower rates of mammogram screening use, which may be influenced by the need for an additional medical visit for the screening. This extra time expenditure can negatively impact one's employment and may inhibit utilization of these services. Single women, including those who are parenting, are generally more dependent on their own sources of income than married women who may have a partner who also provides financial support [40] including access to insurance coverage or perhaps better insurance coverage than they might otherwise have. Further research is needed to examine barriers to obtaining a Pap test among single women.

Prior studies suggest that immigrant women have a lower likelihood of engaging in preventive screenings than U.S.-born women, and this effect is further exacerbated among recently arrived immigrants [11],[14]. In our study, immigrant women who lived in the United States less than 10 years had lower odds of Pap-test utilization than women who lived in the United States for over 15 years, indicating that longer-term acculturation leads to higher utilization rates.

Regarding mammogram utilization, racial/ethnic disparities as well as differences by nativity emerged in the sociodemographic model. Recent statistics showed that Black women now have slightly higher mammography use rates than other women [41], and our study also shows this for Black versus White immigrants. Asian women in the United States are reported to have lower rates of mammogram utilization than White women [41]. In our study, U.S.-born Asian women had higher odds of using mammography screening than their White U.S.-born counterparts in the unadjusted model; however, after adjusting for socioeconomic factors in the full model, that finding remained significant for Asian immigrants only. Since Asian Americans are the most diverse racial group in the United States, and significant socioeconomic variation exists across Asian subgroups [10], more research is needed to examine mammography utilization between and within subgroups by nativity and other acculturation measures. In the full model, socioeconomic and demographic factors had varied effects by women’s nativity. Immigrant women who had lived in the U.S. between 5 and 10 years and U.S-born women with less than a high-school education had lower odds of mammogram utilization than their comparison groups, White immigrants and White U.S. born, respectively. Future studies should further investigate relationships between these factors so that policy and other interventions can be better tailored to reduce socioeconomic, racial/ethnic, and nativity-based disparities in mammogram use.

Our study has the following limitations. Risk factors such as family history of cancer, chronic illnesses, and genetic vulnerability were not available for inclusion. While we excluded women with a history of hysterectomy, we were unable to exclude those with a mastectomy since no question about mastectomy was available in the NHIS. We were also unable to examine generational status among U.S.-born women. Sample size limitations prevented examining the relationship between neighborhood social cohesion and Pap-test and mammogram utilization among ethnic subgroups within immigrant and U.S.-born populations. Participants' data was self-reported, which is subject to recall bias. Although various mechanisms have been hypothesized [18] to explain the relationship between neighborhood social cohesion and preventive services utilization (e.g., social network ties), the data did not allow us to examine them beyond the perceived neighborhood sum score we utilized. Hypotheses related to social network ties and other conceptual formulations of neighborhood social cohesion deserve further examination. Lastly, the cross-sectional data used in this study allow for identification of associations but not causation.

Our study also has strengths. We utilized a large, nationally representative sample and stratified the sample by nativity to examine immigrant and U.S.-born women separately. Given the lack of research examining potential differences between U.S.-born and immigrant persons' utilization of preventive healthcare services, our focus on nativity contributes to filling this gap in the literature. Our study also makes a novel contribution by examining racial/ethnic disparities between groups based on nativity, which allows greater precision in comparing groups.

## 5. Conclusion

Our study expands on the current literature by testing whether perceived neighborhood social cohesion is associated with preventive cancer screenings above and beyond traditional risk factors and control variables. Though we did not find a significant association between neighborhood social cohesion and increased Pap test and mammogram utilization among a nationally representative sample of women in the United States, the socioeconomic and racial/ethnic disparities we did identify indicate the need to target prevention messages and tailor interventions to address each group's sociodemographic characteristics and needs. Researchers should further assess barriers and facilitators of screening use among single women and various immigrant groups. Health insurance was associated with substantially greater odds of Pap test and mammogram cancer screening for immigrant and U.S.-born groups, indicating the need to see that all women have coverage.
