# Correction: Perceived neighborhood social cohesion and cervical and breast cancer screening utilization among U.S.-born and immigrant women

**DOI:** 10.3934/publichealth.2023014

**Published:** 2023-03-08

**Authors:** Quynh Nhu (Natasha) B. La Frinere-Sandoval, Catherine Cubbin, Diana M. DiNitto

**Affiliations:** Steve Hicks School of Social Work, The University of Texas at Austin

**A correction on**

Perceived neighborhood social cohesion and cervical and breast cancer screening utilization among U.S.-born and immigrant women

by Quynh Nhu (Natasha) B, La Frinere-Sandoval, Catherine Cubbin, Diana M. DiNitto. AIMS Public Health, 2022 9(3): 559−573. doi: 10.3934/publichealth.2022039

We would like to submit the following corrections to our recently published paper:

1. The fifth paragraph in section 1 has been updated.

“In the United States, disparities in cervical and breast cancer screening between U.S.-born women and the rapidly growing population of immigrant women call for further examination of social factors, including community and neighborhood factors, that in addition to individual level factors (e.g., income, race/ethnicity, education), may be associated with health behaviors, such as preventive care utilization. Previous research has highlighted both individual and structural factors as important social determinants of health and underlined their relevance for influencing efforts to encourage cancer screening utilization [23]. The Social Determinants of Health conceptual framework [24] illustrates the means by which social, economic and political forces contribute to the socioeconomic stratification of populations based on various factors such as income, gender, employment, education level, marital status, and race/ethnicity. One's socioeconomic status influences these health status drivers since those with low socioeconomic status are generally more susceptible to situations that are harmful to their health. Guided by this conceptual framework, we examined the extent to which neighborhood social cohesion and sociodemographic characteristics influence screening utilization among immigrant and U.S.-born women.”

2. Subsections 2.1 and 2.2 in section 2 have been updated.

*2.1. Data source and study sample*

Data came from the 2018 National Health Interview Survey (NHIS), a nationally representative, cross-sectional household interview survey of the U.S. civilian, non-institutionalized population. NHIS's primary goal is to continuously monitor the U.S. population's health through large scale data collection across a wide spectrum of health issues [25]. The overall sample for this population-based study was the 7801 women ages 21–64 without a hysterectomy. Of them, 7722 (99%) reported Pap test data. The overall sample also included 4211 women ages 40–64 without a hysterectomy, of whom 4087 (78%) reported mammogram data. Of the 7801 women, 1477 (19%) reported being born outside the United States and are considered immigrants. Since virtually all adults age 65 and older in the United States are eligible for Medicare, a federal health insurance program, women in this age group were excluded from the study due to insufficient variance in their insurance status. Participants who identified as belonging to a racial group other than Non-Hispanic Asian, Non-Hispanic Black, Hispanic, or Non-Hispanic White were excluded from our study sample because their numbers were too small for multivariable statistical analyses. The University of Texas at Austin Institutional Review Board reviewed this study's protocol and determined that this is not research involving human subjects and is therefore exempt from IRB oversight.

*2.2. Measures*

Dependent variables were Pap test and mammogram utilization meeting American Cancer Society (ACS) or U.S. Preventive Services Task Force (USPSTF) guidelines. USPSTF recommends that women ages 21−65 of average risk have a Pap test every three years [26]; therefore, we gauged Pap test screening utilization using NHIS's query about “*Most recent Pap test, time categories,”* excluding cases that reported having a hysterectomy. Those reporting that they were screened *“a year ago or less,” “more than 1 year but not more than 2 years,”* or *“more than 2 years but not more than 3 years,”* were coded as “Yes”; the rest were coded as “No.” For mammograms, recommendations are that women ages 40 and older be screened every year [27] or every two years [28]. Using NHIS's query about having had a mammogram “*a year ago or less*” or “*more than 1 year but not more than 2 years,*” we coded those in this age group who responded affirmatively to either query as “Yes” and those who chose another answer as “No.”

The independent variable was perceived neighborhood social cohesion. NHIS queried participants on various neighborhood factors by asking whether they agree or disagree with each of the following four statements using a scale from 1 (definitely agree) to 4 (definitely disagree): 1) “People in this neighborhood help each other out”; 2) “There are people I can count on in this neighborhood”; 3) “People in this neighborhood can be trusted”; and 4) “This is a close-knit neighborhood.” In prior studies, these four items were used to form a neighborhood social cohesion scale that demonstrated high internal consistency (Cronbach's alpha 0.93) [20,29]. Each social cohesion scale item is first reverse coded so that a higher score indicates higher social cohesion; the value of each of the four items is then summed to form a continuous variable with scores ranging from 4 to 16. In our study, we then standardized the summed scores so that in the multivariable analyses the odds ratios indicate neighborhood social cohesion scores as standard deviations from the mean [18]. We imputed any missing or not reported cases for each question separately as the mean of the reported cases for that specific question.

We selected control variables based on previous cancer screening utilization research [15,30,31]. Sociodemographic variables were age (years), marital status (divorced/separated/widowed, never married, married/cohabiting), and race/ethnicity (Asian, Black, Hispanic, White). Socioeconomic status (SES) variables included education (less than high school degree, high school degree, some college, or college degree), employment status (worked last week or not), family income as a share of the federal poverty level (FPL) (FPL <100%, 100–199%, 200–399%, >400%), and health insurance status (uninsured or insured). The Census Bureau defines threshold levels of income based on family size (one or more) and age, adjusted for inflation. This base income level is uniform throughout the United States. Total family income is calculated by summing the incomes of all members of the family. The income for an individual or family can be normalized by transforming it to a percentage of the FPL. Individuals or families with income below 100% FPL are considered the lowest income group and those at or above 400% FPL, are the highest income group [32]. Nativity was defined as U.S.-born vs. immigrant, and acculturation level among immigrants was defined as years living in the United States (less than 5 years, 5–less than 10 years, 10–15 years, and >15 years).

3. Table 1 has been updated.

**Table 1.** Descriptive statistics for women ages 21–64, National Health Interview Survey, 2018, N = 7722.

	Immigrant 1477 (19%)	U.S.-Born 6324 (81%)	T or Chi-Square Statistic	Significance Level
Age (mean)	42.3 (0.4)	40.5 (0.2)	1.77	0.0769
Race/Ethnicity			2616.00	0.0001
Asian	351 (27%)	116 (2%)		
Black	138 (11%)	894 (14%)		
Hispanic	700 (47%)	576 (11%)		
White	274 (16%)	4638 (72%)		
Marital Status			63.54	0.0001
Divorced/Separated/Widowed	271 (13%)	1280 (13%)		
Never Married	267 (15%)	1696 (26%)		
Married/Cohabiting	937 (72%)	3335 (61%)		
Education			356.63	0.0001
Less than high school degree	304 (20%)	386 (6%)		
High school degree	304 (22%)	1216 (20%)		
Some college	291 (20%)	2077 (33%)		
College graduate	569 (38%)	2631 (41%)		
Employment Status			28.64	0.0001
Did not work last week	548 (39%)	1895 (30%)		
Worked last week	927 (61%)	4427 (70%)		
Income (% of Federal Poverty Level)			101.33	0.0001
<100%	293 (17%)	864 (11%)		
100%–199%	341 (24%)	1,017 (15%)		
200%–299%	212 (15%)	944 (15%)		
300%–399%	163 (11%)	850 (14%)		
>400%	468 (32%)	2649 (45%)		
Health Insurance			152.94	0.0001
Not covered	307 (21%)	593 (9%)		
Covered	1164 (79%)	5708 (91%)		
Years living in U.S.				
<5 years	182 (12%)			
5–less than 10 years	141 (11%)			
10–15 years	183 (12%)			
>15 years	950 (64%)			
Perceived neighborhood social cohesion			
	11.9 (0.1)	12.4 (0.1)	−5.13	0.0001
Pap-test last 3 years (ages 21–64)			21.31	0.0001
Yes	1120 (76%)	5120 (82%)		
No	348 (24%)	1170 (18%)		
Mammogram last 2 years (ages 40–64)			2.04	0.1537
Yes	514 (62%)	2168 (66%)		
No	312 (38%)	1093 (34%)		

4. Updated the subtitle of section 3 by deleting 3.1.

*Participants' characteristics*

5. The third paragraph and the rest of section 3 have been updated.

“Racial/ethnic disparities emerged in the sociodemographic models for Pap test utilization. Both U.S.-born and immigrant Hispanic women and U.S.-born Black women had higher odds of having a Pap test than their White counterparts. Other results were similar to the unadjusted models. An additional racial/ethnic disparity emerged in the full model with immigrant Asian women having lower odds of Pap test use than immigrant White women. Other results were similar to the unadjusted and sociodemographic models, except that for U.S.-born women, being previously married or unemployed was no longer statistically significant. For both groups, older age was associated with lower odds of getting a Pap test.

Table 3 presents odds ratios and confidence intervals for mammogram utilization (for women ages 40–64).

In the unadjusted models, among immigrant women, those who had less than a high-school education, or income lower than 200% had significantly lower odds of mammogram utilization, while among U.S.-born women, those who were Asian, were previously or never married, had high-school degree or less education, were unemployed, or had income lower than 400% had lower odds of having a mammogram.

As with Pap test utilization, racial/ethnic disparities emerged in the sociodemographic model. Both U.S.-born and immigrant women who lacked insurance had lower odds of mammogram utilization. Immigrant Black women and Asian women had higher odds of mammogram utilization than their White counterparts. Among immigrant women, those who had lived in the United States for less than 10 years had lower odds of having a mammogram than those living in the United States for more than 15 years. Among U.S.-born women, those who were Asian and those who never married had lower odds of mammogram utilization. Perceived social cohesion was associated with higher odds of mammogram utilization among U.S.-born women (OR = 1.63, CI = 1.02, 2.60).

Most of the significant factors remained in the full model. For both immigrant and U.S.-born groups, older age was associated with higher odds of mammogram utilization, while not having insurance coverage and income less than 200% was associated with lower odds. Among immigrants, Black and Asian (compared with White) women had *higher* odds of mammogram utilization. Those who had lived in the United States for less than 10 years had lower odds of having mammogram utilization than those living in the United States for more than 15 years. Continuing the same trend, among U.S.-born women, Black women had higher odds of mammogram utilization compared to their White counterparts. Those who had less than a high school degree had lower odds of mammogram utilization. For both groups, perceived social cohesion had no effect.”

6. Title of Table 2 has been updated.

**Table 2.** Odds ratios of Pap test utilization, NHIS, U.S., 2018, N = 7722

7. Table 3 has been updated.

**Table 3.** Odds ratios of mammogram utilization, NHIS, U.S., 2018, N = 4087

	Unadjusted Model				Sociodemographic Models				Full Models			
	Immigrant		U.S.-Born		Immigrant		U.S.-Born		Immigrant		U.S.-Born	
	O.R.	95% C.I.	O.R.	95% C.I.	O.R.	95% C.I.	O.R.	95% C.I.	O.R.	95% C.I.	O.R.	95% C.I.
Age	1.05	[1.03, 1.08]	1.04	[1.03, 1.05]	1.04	[1.02, 1.07]	1.04	[1.03, 1.06]	1.05	[1.02, 1.07]	1.05	[1.04, 1.06]
Race/Ethnicity												
Asian	1.01	[0.69, 1.47]	0.57	[0.42, 0.77]	1.75	[1.13, 2.70]	0.71	[0.51, 0.98]	2.87	[1.70, 4.85]	0.80	[0.57, 1.13]
Black	1.32	[0.73, 2.41]	0.87	[0.67, 1.13]	2.15	[1.12, 4.11]	1.06	[0.80, 1.40]	2.83	[1.38, 5.80]	1.31	[1.00, 1.72]
Hispanic	0.87	[0.57, 1.33]	1.24	[0.49, 3.13]	0.92	[0.59, 1.42]	1.37	[0.56, 3.35]	0.97	[0.62, 1.51]	1.11	[0.45, 2.76]
White	1.00		1.00		1.00		1.00		1.00		1.00	
Marital Status												
Divorced/Separated/Widowed	0.96	[0.67, 1.36]	0.80	[0.67, 0.96]	0.75	[0.51, 1.11]	0.84	[0.69, 1.01]	0.93	[0.62, 1.42]	1.02	[0.83, 1.25]
Never Married	0.75	[0.44, 1.27]	0.58	[0.46, 0.73]	0.67	[0.37, 1.20]	0.64	[0.49, 0.83]	0.79	[0.42, 1.47]	0.77	[0.59, 1.01]
Married/Cohabiting	1.00		1.00		1.00		1.00		1.00		1.00	
Education												
Less than high school degree	0.50	[0.34, 0.74]	0.39	[0.29, 0.53]					0.69	[0.41, 1.16]	0.69	[0.48, 0.99]
High school degree	0.69	[0.47, 1.02]	0.59	[0.48, 0.74]					0.72	[0.43, 1.18]	0.76	[0.59, 0.98]
Some college	0.89	[0.57, 1.37]	0.68	[0.56, 0.82]					0.98	[0.60, 1.60]	0.76	[0.61, 0.95]
College graduate	1.00		1.00						1.00		1.00	
Employment Status												
Did not work last week	0.87	[0.66, 1.16]	0.74	[0.63, 0.87]					1.11	[0.79, 1.57]	0.83	[0.68, 1.00]
Worked last week	1.00		1.00						1.00		1.00	
Income (% of Federal Poverty Level)											
<100%	0.32	[0.21, 0.51]	0.28	[0.21, 0.36]					0.35	[0.18, 0.65]	0.42	[0.30, 0.58]
100%–199%	0.45	[0.30, 0.68]	0.44	[0.34, 0.56]					0.48	[0.28, 0.84]	0.60	[0.44, 0.82]
200%–299%	0.64	[0.37, 1.11]	0.63	[0.49, 0.81]					0.62	[0.33, 1.14]	0.77	[0.59, 1.01]
300%–399%	0.56	[0.32, 1.00]	0.72	[0.56, 0.94]					0.56	[0.29, 1.09]	0.79	[0.60, 1.04]
≥400%	1.00		1.00						1.00		1.00	
Health Insurance Coverage												
Not covered	0.26	[0.17, 0.39]	0.23	[0.18, 0.30]	0.23	[0.14, 0.36]	0.23	[0.17, 0.31]	0.27	[0.16, 0.43]	0.28	[0.21, 0.37]
Covered	1.00		1.00		1.00		1.00		1.00		1.00	
Years Living in U.S.												
<5 years	0.36	[0.18, 0.73]			0.48	[0.24, 0.95]			0.49	[0.24, 1.01]		
5–less than 10 years	0.36	[0.17, 0.77]			0.36	[0.16, 0.84]			0.41	[0.17, 0.97]		
10–15 years	0.79	[0.47, 1.32]			0.91	[0.53, 1.57]			1.03	[0.59, 1.79]		
>15 Years	1.00				1.00				1.00			
Perceived Neighborhood Social Cohesion											
	0.68	[0.29, 1.63]	1.70	[1.09, 2.65]	0.81	[0.32, 2.08]	1.63	[1.02, 2.60]	0.80	[0.30, 2.12]	1.27	[0.79, 2.04]
Perceived Neighborhood Social Cohesion Squared										
	1.73	[0.71, 4.20]	0.69	[0.44, 1.07]	1.37	[0.53, 3.54]	0.69	[0.43, 1.10]	1.37	[0.51, 3.69]	0.83	[0.52, 1.33]

8. The third paragraph in section 4 has been updated.

“The sociodemographic model also revealed racial/ethnic disparities in Pap test utilization that are contrary to commonly reported trends. For example, like some previous studies, we found that among both U.S.-born and immigrant groups, Hispanic women had higher odds of Pap test use than White women [36] and that among the U.S.-born, Black women had higher odds of Pap test use than White women [37]. Any obstacles these groups may have faced in obtaining preventive services might have been overcome through increased access and outreach. For example, national and regional programs and initiatives have been launched that are specifically tailored to racial minority populations in an effort to reduce disparities and improve cancer screening among these groups [37]. In the full model for immigrant women, consistent with other studies [11,38], another racial/ethnic disparity emerged in that Asian women had lower odds of Pap test screenings than their White counterparts. However, pooling NHIS data from 4 years (2005, 2008, 2013, 2015), Endeshaw et al. [39] found that the likelihood of having received a Pap test within 3 years for immigrant Southeast Asian women was comparable to U.S.-born women. Although those results suggest that Pap test utilization has increased in recent years among Asian immigrant women, our study indicates that this group remains at risk of underutilization of cervical cancer preventive screenings. In comparing Asian immigrants to White immigrants, we found that disparities in utilization persist.”

9. The sixth paragraph in section 4 has been updated.

“Regarding mammogram utilization, racial/ethnic disparities as well as differences by nativity emerged in the sociodemographic model. Recent statistics showed that Black women now have slightly higher mammography use rates than other women [41], and our study also shows this for Black versus White immigrants. Asian women in the United States are reported to have lower rates of mammogram utilization than White women [41]. In our study, U.S.-born Asian women had higher odds of using mammography screening than their White U.S.-born counterparts in the unadjusted model; however, after adjusting for socioeconomic factors in the full model, that finding remained significant for Asian immigrants only. Since Asian Americans are the most diverse racial group in the United States, and significant socioeconomic variation exists across Asian subgroups [10], more research is needed to examine mammography utilization between and within subgroups by nativity and other acculturation measures. In the full model, socioeconomic and demographic factors had varied effects by women's nativity. Immigrant women who had lived in the U.S. between 5 and 10 years and U.S-born women with less than a high-school education had lower odds of mammogram utilization than their comparison groups, White immigrants and White U.S. born, respectively. Future studies should further investigate relationships between these factors so that policy and other interventions can be better tailored to reduce socioeconomic, racial/ethnic, and nativity-based disparities in mammogram use.”

